# A comparative review of inflammatory bowel disease guidelines: JSGE, ECCO, AGA, and ACG

**DOI:** 10.17179/excli2026-9575

**Published:** 2026-09-29

**Authors:** Yuta Shimomori, Yoshihiro Yokoyama, Kotaro Akita, Tomoe Kazama, Kohei Wagatsuma, Kazuhiro Mizukami, Hiroshi Nakase

**Affiliations:** 1 Division of Gastroenterology and Hepatology, Department of Internal Medicine, Sapporo Medical University, S-1, W-16, Chuo-ku, Sapporo 060-8543, Hokkaido, Japan; 2 Department of Gastroenterology, Faculty of Medicine, Oita University, 1-1 Idaigaoka, Hasama, Yufu 879-5593, Oita, Japan

**Keywords:** inflammatory bowel disease, ulcerative colitis, Crohn's disease, guideline comparison

## Abstract

Inflammatory bowel disease (IBD), encompassing ulcerative colitis (UC) and Crohn's disease (CD), is managed according to clinical practice guidelines that integrate evidence appraisal with regional healthcare contexts. This narrative, comparative review examined selected guideline documents from the Japanese Society of Gastroenterology (JSGE), European Crohn's and Colitis Organisation (ECCO), American Gastroenterological Association (AGA), and American College of Gastroenterology (ACG) using the JSGE guidelines as the organizing framework to highlight similarities and differences in recommendation direction and contextual nuance. The analysis focused on the guideline architecture and selected domains of monitoring and medical therapy, with particular attention to how document structure, scope, publication timing, and update strategies shape the interpretation of similarities and differences in recommendation direction. Across the guideline systems, general agreement in overall direction was observed regarding the shift from symptom-based assessment alone towards objective monitoring, the sustained role of 5-aminosalicylic acid (5-ASA) in the treatment of UC, the expanding integration of advanced therapies, and the adoption of risk-based surveillance principles for IBD-associated colorectal neoplasia. The most notable divergence concerned the use of 5-ASA in CD treatment: the JSGE guidelines retained a more pragmatic role for this therapy, whereas the recent ECCO, AGA, and ACG guidelines generally limited routine use. Treatment withdrawal and de-escalation emerged as particularly heterogeneous domains, reflecting the uneven treatment of drug-specific recommendations. The JSGE guidelines were notable for their distinctive single-volume integration of diagnostic criteria, treatment strategy, and follow-up, as well as their explicit inclusion of Japan-specific considerations, such as nucleoside diphosphate-linked moiety X-type motif 15 genotyping, cytapheresis, and oral tacrolimus. These findings suggest that apparent differences among IBD guidelines should be understood not only as differences in clinical judgment, but also as products of guideline architecture, update timing, and intended scope.

See also the graphical abstract(Fig. 1).

## 1. Introduction

Inflammatory bowel disease (IBD), encompassing ulcerative colitis (UC) and Crohn's disease (CD), is a chronic relapsing-remitting inflammatory disorder of the gastrointestinal tract (Nakase et al., 2021[9]). The prevalence of IBD has recently increased worldwide, including in Japan (Heydari et al., 2025[3]; Tsutsui et al., 2025[15]). IBD care is also shaped by regional differences in healthcare systems, drug availability, reimbursement frameworks, monitoring practices, and treatment strategies.

Against this background, IBD guidelines differ not only in their recommendations but also in their structure and update processes. Major societies in Japan, Europe, and the United States of America (U.S.) have developed influential guidelines for UC and CD. The Japanese Evidence-based Clinical Practice Guidelines for IBD 2020 provide comprehensive guidance for both UC and CD (Nakase et al., 2021[9]). In contrast, the European and U.S. guideline systems more often distribute clinical guidance across modular, disease-specific, or topic-specific documents, with periodic or living updates in selected areas (Ananthakrishnan et al., 2023[1]; Gordon et al., 2024[2]; Ko et al., 2019[5]; Kucharzik et al., 2025[6]; Lichtenstein et al., 2025[7]; Raine et al., 2022[10]; Rubin et al., 2025[11]; Scott et al., 2025[12]; Singh et al., 2023[13], 2024[14]; Yanai et al., 2025[17]). These structural differences affect how the recommendations are framed, updated, and interpreted.

This review compared selected guidelines from the Japanese Society of Gastroenterology (JSGE), European Crohn's and Colitis Organisation (ECCO), American Gastroenterological Association (AGA), and American College of Gastroenterology (ACG), using the JSGE guidelines as structural anchors. Particular attention was given to monitoring, medical therapy, and the interpretation of apparent similarities and differences in recommendation direction across different guideline systems.

## 2. Structure of the Japanese IBD Guideline Framework

The Japanese Evidence-based Clinical Practice Guidelines for IBD 2020 are organized as an integrated framework for both UC and CD. These guidelines address disease concepts, diagnostic criteria, clinical assessment, severity classification, treatment strategies, complications, special situations, and longitudinal management (Nakase et al., 2021[9]). This structure frames UC and CD care as a continuous clinical pathway from diagnosis through initial treatment selection to follow-up.

The guidelines are organized around background questions (BQs), clinical questions (CQs), and future research questions (FRQs). This question-based format allows for the establishment of background knowledge, actionable clinical recommendations, and areas requiring further evidence to be presented within the same framework (Figure 2(Fig. 2)).

## 3. Methods of Comparative Review

This study was a narrative, comparative review of selected clinical practice guidelines for IBD. The 2020 Evidence-based Clinical Practice Guidelines for IBD issued by the JSGE served as the organizing framework for the comparison because they provide a comprehensive, single-volume approach to diagnosis, medical management, and longitudinal follow-up across the continuum of care (Nakase et al., 2021[9]).

Comparator documents were identified from the official websites of ECCO, AGA, and ACG, based on the guideline documents available as of May 2026. For each guideline system, the most recent formally designated clinical practice guideline addressing the selected comparison domains of monitoring or medical therapy was included. When an updated guideline was available for the same clinical topic, the earlier version was excluded. Other society documents were considered at the title and scope level but were not included if they were consensus statements, position statements, expert reviews, clinical practice updates, or other publications not formally designated as clinical practice guidelines, or if they primarily addressed topics outside the selected comparison domains.

Twelve prespecified guideline documents were included in this comparative review, consisting of the JSGE guideline as the reference framework and 11 comparator documents from ECCO, AGA, and ACG (Table 1(Tab. 1)) (Ananthakrishnan et al., 2023[1]; Gordon et al., 2024[2]; Ko et al., 2019[5]; Kucharzik et al., 2025[6]; Lichtenstein et al., 2025[7]; Nakase et al., 2021[9]; Raine et al., 2022[10]; Rubin et al., 2025[11]; Scott et al., 2025[12]; Singh et al., 2023[13], 2024[14]; Yanai et al., 2025[17]). The number of comparator documents differed among the societies because their guideline systems differ in how clinical guidance is organized across comprehensive, disease-specific, or topic-specific documents; the source set was therefore selected to provide coverage of the predefined comparison domains rather than to achieve numerical balance across organizations.

In this review, we compared selected guideline documents with respect to their structure and recommendations rather than reassessing or comparing the primary studies that informed individual recommendations. Primary studies, systematic reviews, and external consensus frameworks were cited only when necessary to contextualize differences in guideline recommendations or their regional clinical background and were not independently appraised as part of the comparative analysis.

The analysis was limited to prespecified domains related to monitoring and medical therapy. Similarities and differences in recommendation direction across guidelines were assessed qualitatively, considering the regional practice context, publication date, document scope, topic allocation, update structure, and guideline development methodology. General agreement in overall direction was considered present when the included guidelines supported a broadly similar clinical approach, even when the strength, wording, target population, or operational details of the recommendations differed. Directional differences were identified when the guidelines supported substantively different clinical approaches. Domains in which corresponding recommendations were absent or not directly comparable because of differences in scope or document structure were considered heterogeneous.

Surgical, postoperative, and colorectal neoplasia management beyond surveillance were not selected as primary comparison domains because their scope, document placement, and depth of coverage differ substantially across the included guideline sets. Including these areas as equivalent comparison domains could have led to misleading conclusions, because apparent differences would often reflect differences in document structure rather than true differences in recommendation direction.

## 4. Guideline Architecture and Sources of Divergence

Although the included guidelines are directed toward broadly overlapping clinical audiences, they differ substantially in how guidance is organized, distributed, and updated. These structural features are not merely editorial distinctions; they influence which recommendations are visible, how broadly they apply, and how current they remain. Accordingly, we treated these differences as methodologically relevant when interpreting apparent similarities and differences in recommendation direction across guideline systems. Table 2(Tab. 2) summarises the major systems according to document configuration, scope, and update strategy.

The JSGE guidelines served as the structural anchor for this review because they address the principal domains of IBD care for both UC and CD within a single integrated framework (Nakase et al., 2021[9]).

By contrast, ECCO issues its guidance through multiple modular documents. Recommendations regarding therapy, diagnosis, and monitoring are therefore distributed across related but separate publications, which, in some instances, differ in their development processes and methods (Gordon et al., 2024[2]; Kucharzik et al., 2025[6]; Raine et al., 2022[10]; Yanai et al., 2025[17]). This structure permits greater domain-specific depth; however, no single ECCO document necessarily captures ECCO's full position on IBD care.

AGA is likewise presented as a series of topic-specific documents, but it follows a different organizing logic. Among the sources selected for this review, separate AGA documents address the management of mild-to-moderate UC, the use of biomarker guidance for UC and CD, and living pharmacological recommendations for moderate-to-severe disease (Ananthakrishnan et al., 2023[1]; Ko et al., 2019[5]; Scott et al., 2025[12]; Singh et al., 2023[13], 2024[14]). This format facilitates focused updates within specific clinical domains but also distributes related recommendations across documents that vary in scope and purpose.

ACG guidance follows a disease-specific integrative model. The UC and CD guidelines bring together major clinical domains within a single disease-oriented volume, whereas the selected subdomains remain less fully developed than in a dedicated topic-specific set (Lichtenstein et al., 2025[7]; Rubin et al., 2025[11]). Thus, the ACG guidance provides greater integration within individual documents than a purely topic-based model; however, it does not offer a single unified architecture across the full IBD guideline corpus.

Taken together, these structural differences suggest that the prominence and granularity of recommendations may reflect both the distribution of content across documents and substantive differences in guideline positions. Accordingly, this section focuses on monitoring and medical therapy as the principal comparative domains for assessing the direction of recommendations more consistently across guideline systems.

## 5. Similarities, Differences, and Heterogeneity across Guideline Recommendations

This section is not intended to formally classify the included guidelines as concordant or discordant, but rather to distinguish domains showing general agreement in overall direction, clearer directional differences, and heterogeneity in which direct comparison across source documents is limited. This distinction is especially important because the JSGE guidelines are comprehensive documents, whereas the ECCO, AGA, and ACG materials are organized as modular, topic-specific, or disease-specific source sets (Figure 3(Fig. 3)).

### 5.1. General agreement in overall direction

General agreement in overall direction was observed regarding the need for objective monitoring. Across the included guideline sets, symptoms alone are no longer considered sufficient for contemporary IBD management; biomarker- and endoscopy-informed assessments are increasingly incorporated into clinical decision-making (Ananthakrishnan et al., 2023[1]; Kucharzik et al., 2025[6]; Lichtenstein et al., 2025[7]; Nakase et al., 2021[9]; Rubin et al., 2025[11]; Singh et al., 2023[13]; Yanai et al., 2025[17]). However, the presentation and operationalization of this approach differ across guideline systems. In the JSGE guidelines, objective monitoring is embedded within a comprehensive framework, whereas the European and U.S. sources make this domain more explicit through dedicated diagnostic and biomarker documents, particularly in the ECCO diagnostics and monitoring guidance and the AGA biomarker guidelines (Ananthakrishnan et al., 2023[1]; Kucharzik et al., 2025[6]; Singh et al., 2023[13]; Yanai et al., 2025[17]). Thus, the general agreement in overall direction lies not in complete uniformity regarding the recommended use of each monitoring modality, but in a shared movement away from symptom-only assessment. This overall direction is also consistent with the STRIDE II treat-to-target framework, which emphasizes objective treatment targets, including biomarker normalization and endoscopic healing, in contemporary IBD care (Turner et al., 2021[16]). STRIDE II is an external consensus framework and was not included among the clinical practice guideline documents compared in this review.

A second area showing general agreement in overall direction concerns the role of 5-aminosalicylic acid (5-ASA) in UC. Despite differences in document structure, the included guidelines consistently preserve 5-ASA as a core therapeutic option, especially for mild-to-moderate disease, and continue to support combined oral and rectal use in appropriately selected patients (Ko et al., 2019[5]; Nakase et al., 2021[9]; Raine et al., 2022[10]; Rubin et al., 2025[11]).

A similar overall direction is evident in the expanding role of advanced therapies. Across guideline systems, the overall direction of treatment has shifted towards earlier and more structured use of biologics and small molecules, although the formal positioning of newer agents is more explicit in recent living or frequently updated European and U.S. guideline documents (Gordon et al., 2024[2]; Lichtenstein et al., 2025[7]; Raine et al., 2022[10]; Rubin et al., 2025[11]; Scott et al., 2025[12]; Singh et al., 2024[14]).

Another area showing general agreement in overall direction concerns surveillance in patients at an increased risk of IBD-associated colorectal neoplasia. The included documents generally concur that long-standing colitis and relevant risk factors warrant structured colonoscopic surveillance, with surveillance intensity tailored to the patient's risk profile (Kucharzik et al., 2025[6]; Nakase et al., 2021[9]; Yanai et al., 2025[17]). In this domain, similarity in overall direction is clearer at the level of overarching surveillance principles than in the details of downstream management.

### 5.2. Directional differences and heterogeneous domains

The clearest divergence concerns the use of 5-ASA in CD. In contrast to its established role in UC, the JSGE guidelines retain a practical role for 5-ASA in CD, whereas recent ECCO, AGA, and ACG guidelines generally limit or do not support its routine use for luminal CD (Ananthakrishnan et al., 2023[1]; Gordon et al., 2024[2]; Lichtenstein et al., 2025[7]; Nakase et al., 2021[9]).

Treatment de-escalation and withdrawal are best viewed as a heterogeneous domain, rather than as a domain showing a uniform pattern of agreement or difference in recommendation direction. Across guideline systems, rapid tapering and discontinuation of systemic corticosteroids are consistently emphasized, whereas the withdrawal of advanced therapies is approached more cautiously because of the risk of relapse (Gordon et al., 2024[2]; Ko et al., 2019[5]; Lichtenstein et al., 2025[7]; Nakase et al., 2021[9]; Raine et al., 2022[10]; Rubin et al., 2025[11]; Scott et al., 2025[12]; Singh et al., 2024[14]). Among the selected sources, the explicitness with which this topic is addressed varies. Recent European and U.S. guidelines discuss specific withdrawal scenarios more directly, including discontinuation of anti-TNF therapy and withdrawal of the immunomodulator component of combination therapy in CD, whereas the JSGE guidelines also frame biological withdrawal as an issue requiring careful clinical discussion (Gordon et al., 2024[2]; Nakase et al., 2021[9]; Scott et al., 2025[12]). Discontinuation of 5-ASA after escalation to advanced therapy is addressed most explicitly in some European and U.S. guidelines for UC (Rubin et al., 2025[11]; Singh et al., 2024[14]). A comparable recommendation is less apparent for CD, in which 5-ASA already plays a limited therapeutic role and is not clearly articulated in the JSGE guidelines. Overall, this domain shows some similarity in overall direction regarding the need for caution but uneven comparability in drug-specific recommendations.

Taken together, the included guideline sets show greater similarity in overall recommendation direction than a simple contrastive reading might suggest. The most defensible interpretation is that clear differences in recommendations are confined to selected domains, most notably 5-ASA in CD, whereas many other apparent differences reflect unequal visibility, update timing, or distribution of guidance across documents.

## 6. Japan-Specific Perspectives beyond the Core Comparison Domains

In addition to the core comparison domains discussed above, the JSGE guidelines highlight several diagnostic, safety-related, and therapeutic features that are particularly prominent in Japanese IBD practices. This section summarises selected perspectives specific to the Japanese guideline framework (Figure 4(Fig. 4)).

One notable feature is the explicit presentation of the diagnostic criteria for UC and CD within the JSGE guideline framework (Nakase et al., 2021[9]). Since the diagnosis of IBD requires the integration of clinical symptoms, endoscopic findings, histologic features, imaging results, and the exclusion of disease mimics, these criteria provide a practical basis for disease classification and subsequent management. This diagnostic framework is also relevant to IBD-unclassified (IBDU), a category applied to patients who cannot be clearly classified as having typical UC or CD at the initial assessment.

Another important example is the prominent recommendation for pre-thiopurine nucleoside diphosphate-linked moiety X-type motif 15 (NUDT15) genotyping. In Japan, NUDT15 is explicitly incorporated into pretreatment safety assessments because relevant variants are strongly associated with severe leukopenia and alopecia during thiopurine therapy. This pharmacogenetic issue is less prominently emphasised in the included European and U.S. guidelines (Kakuta et al., 2018[4]; Nakase et al., 2021[9]; Yoichi et al., 2016[18]).

A similar distinction applies to the continued visibility of cytapheresis and oral tacrolimus in the JSGE framework (Nakase et al., 2021[9]). In Japan, granulocyte-monocyte adsorptive apheresis has been evaluated extensively in domestic clinical studies and remains a recognizable therapeutic option within the Japanese UC treatment framework (Naganuma et al., 2020[8]; Yoshino et al., 2014[19]). Its prominence is lower in the included European and U.S. guideline documents. Oral tacrolimus is similarly retained as an induction option for UC in the JSGE guideline, whereas its role is more limited in the included European and U.S. guidance. These supporting studies are cited here to contextualize the regional visibility of these therapies rather than to independently assess their efficacy.

Taken together, these examples indicate that the JSGE guidelines are shaped not only by the appraisal of the international evidence base but also by domestic clinical data, therapeutic practices, and safety considerations that remain directly relevant to clinical care in Japan.

## 7. Limitations

This study has several limitations. First, it was designed as a narrative comparison of selected guidelines rather than as a systematic review of the underlying primary studies. Accordingly, the analysis focused on how recommendations and guideline structures are presented rather than on the independent reassessment of the evidence base supporting each recommendation.

Second, the included guideline systems differ substantially in document structure, scope, timing of publication, and approaches to updating. These differences limit direct one-to-one comparisons and raise the possibility that some apparent differences reflect the allocation of content across documents or differences in update cadence, rather than true disagreement in clinical principles. This limitation is particularly relevant for rapidly evolving domains such as advanced therapies: the JSGE guideline was published in 2020, whereas several European and U.S. documents were updated in 2024 or 2025.

Third, the review focused primarily on monitoring and medical therapy. Surgical care, postoperative management, and the management of colorectal neoplasia beyond surveillance were not compared in comparable depth, because these topics differed substantially across guideline systems in scope, placement, and level of detail. Diagnostic entities such as IBDU were addressed descriptively rather than as independent comparative domains. Similarly, diagnostic considerations emphasized in Japanese clinical practice, including MEFV-associated enterocolitis, were not included in formal domain-level comparisons because they are not uniformly addressed across the included guideline documents.

## 8. Conclusion

This review highlights three principal findings. First, the selected IBD guidelines show general agreement in overall direction across several major domains, including objective monitoring, the use of 5-ASA in UC, incorporation of advanced therapies, and surveillance principles for IBD-associated colorectal neoplasia. Second, many apparent differences among the guidelines reflect differences in document structure, timing of publication, and allocation of scope, rather than direct disagreement in clinical judgment. Third, the JSGE guidelines are distinctive in that they combine nationally defined diagnostic criteria with integrated pathways for UC/CD, spanning diagnosis, treatment, and follow-up within a single clinical document. This integrated structure provides a useful framework for understanding the overall continuum of IBD care, whereas the European and U.S. guidelines often provide greater depth within specific domains through modular, disease-specific, topic-specific, or living documents.

Guidelines should, therefore, be understood not only as evidence-based recommendations but also as documents shaped by structure, update timing, and intended scope. Clinicians should use these frameworks for decision-making while adapting recommendations to each patient's clinical background, disease phenotype, and treatment goals.

## Declaration

### Acknowledgments

None.

### Conflict of interest

The authors declare that they have no conflicts of interest.

### Artificial Intelligence (AI) - assisted technology

No artificial intelligence (AI)-assisted technologies (e.g., Large Language Models [LLMs], chatbots, or image creations) were used in the submission process to complete the manuscript.

### Author contributions

YS, YY, KA, TK, KW, KM, and HN contributed to methodology. YS, YY, and HN contributed to conceptualization, data curation, and formal analysis. YS and YY contributed to investigation and visualization. YS contributed to writing - original draft. HN contributed to supervision, writing - review & editing.

## Figures and Tables

**Table 1:**
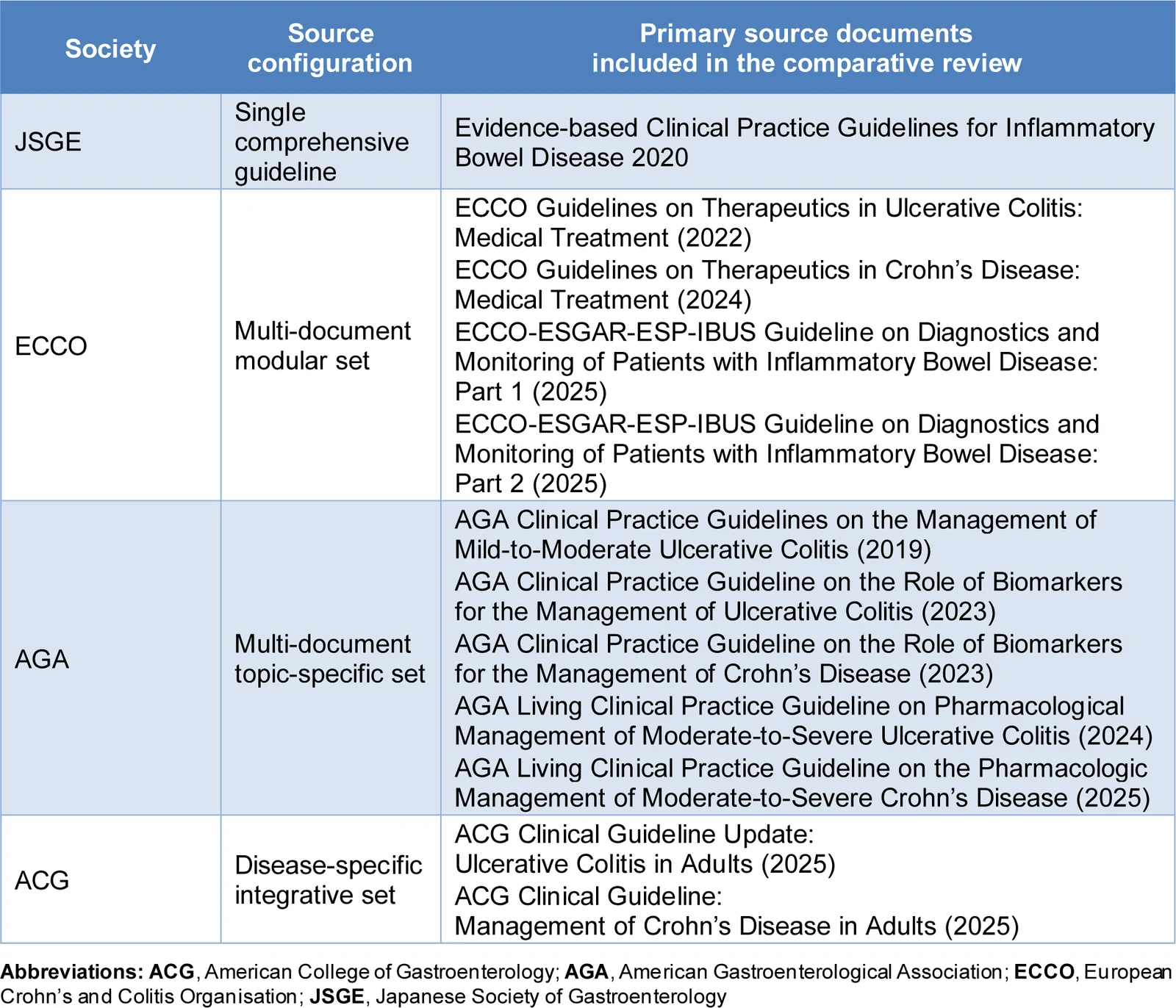
Source documents included in the comparative review

**Table 2:**
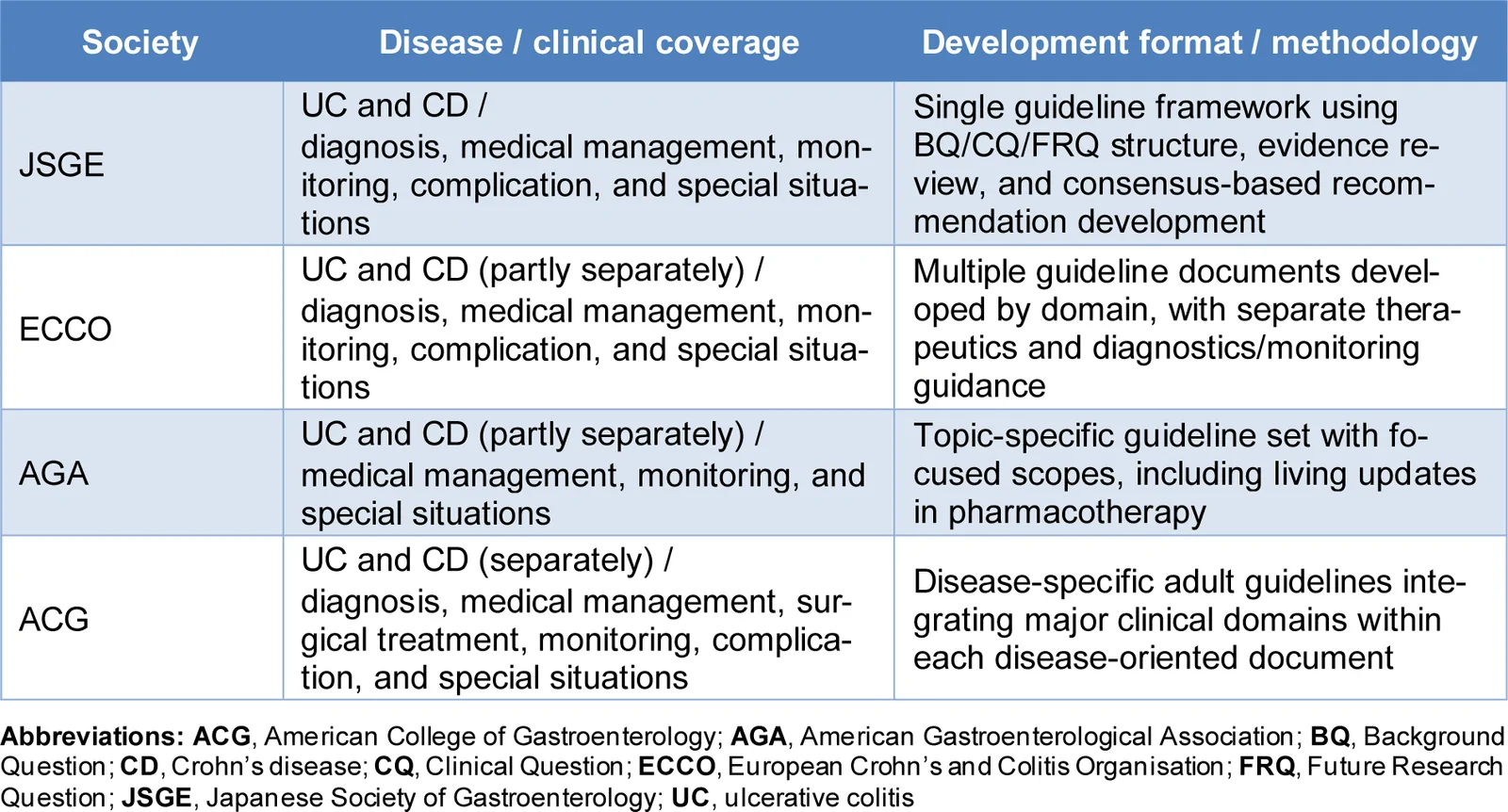
Comparative architecture of JSGE, ECCO, AGA, and ACG guideline systems

**Figure 1:**
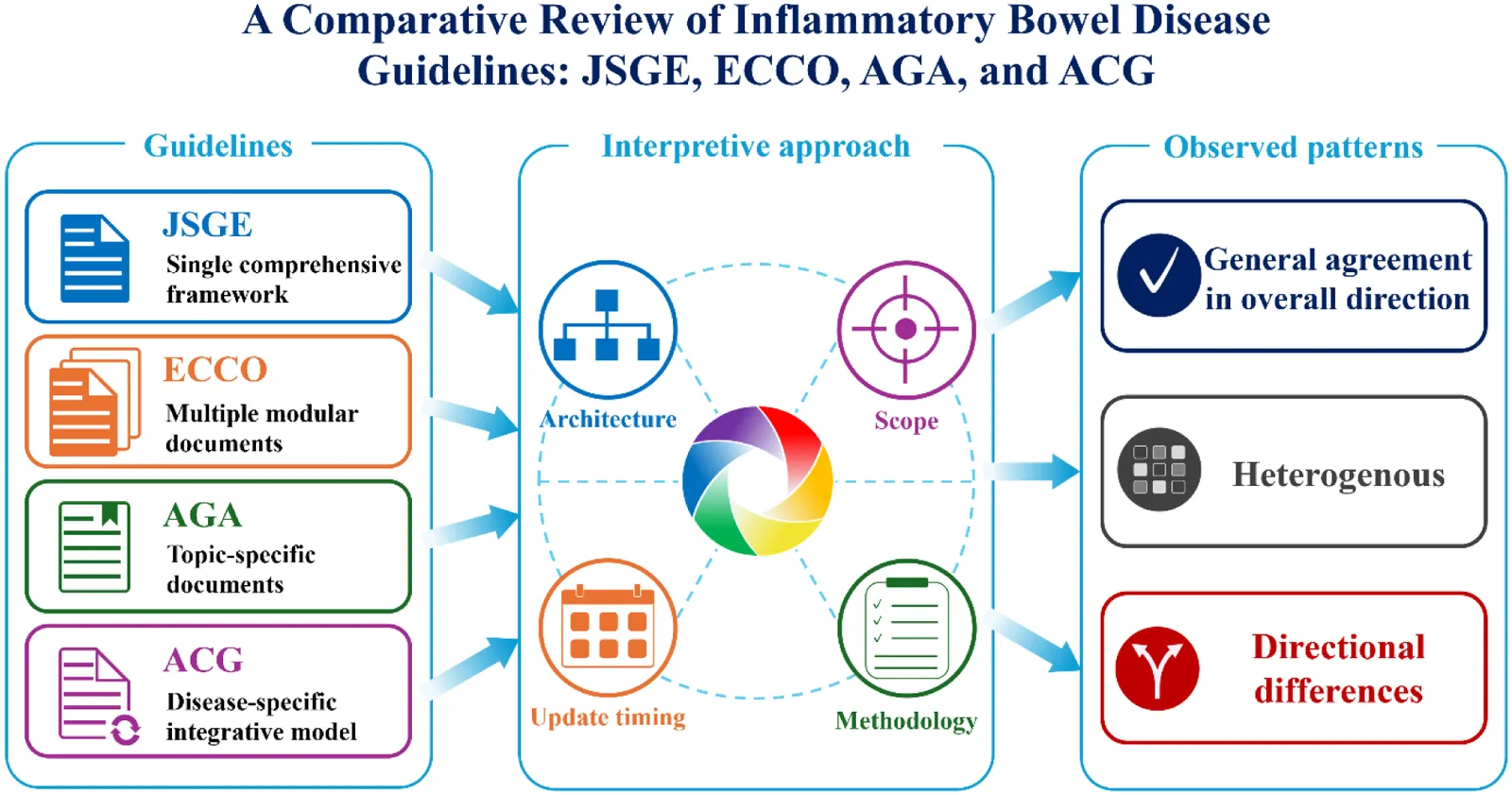
Graphical abstract

**Figure 2:**
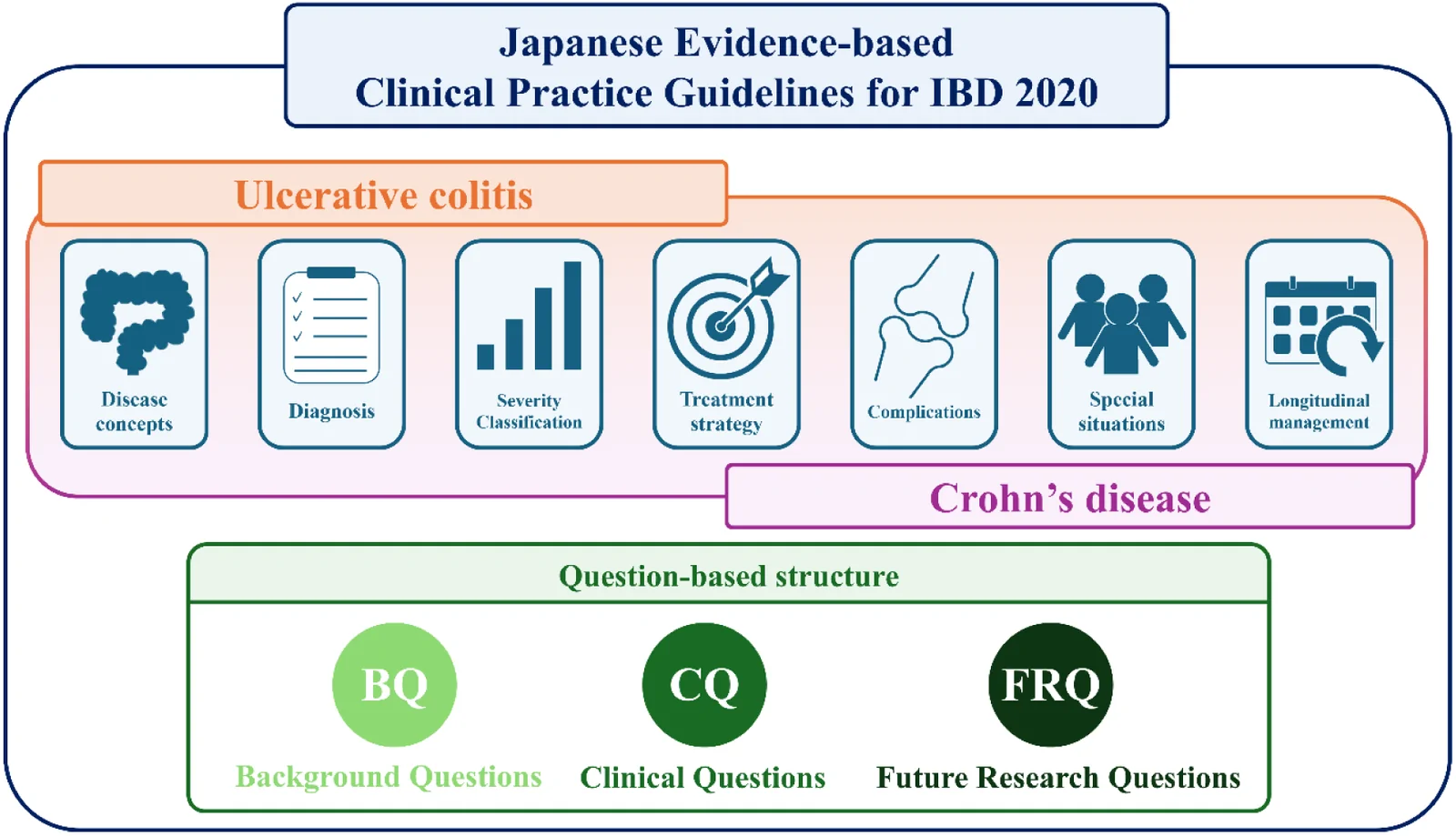
Integrated structure of the Japanese IBD guideline framework. The Japanese Evidence-based Clinical Practice Guidelines for IBD 2020 cover both UC and CD within a single integrated framework. The guideline spans diagnosis, severity classification, treatment selection, complications and special situations, and longitudinal follow-up, and is organized through BQs, CQs, and FRQs. Abbreviations: hichBQ, Background Question; CD, Crohn's disease; CQ, Clinical Question; FRQ, Future Research Question; IBD, inflammatory bowel disease; UC, ulcerative colitis

**Figure 3:**
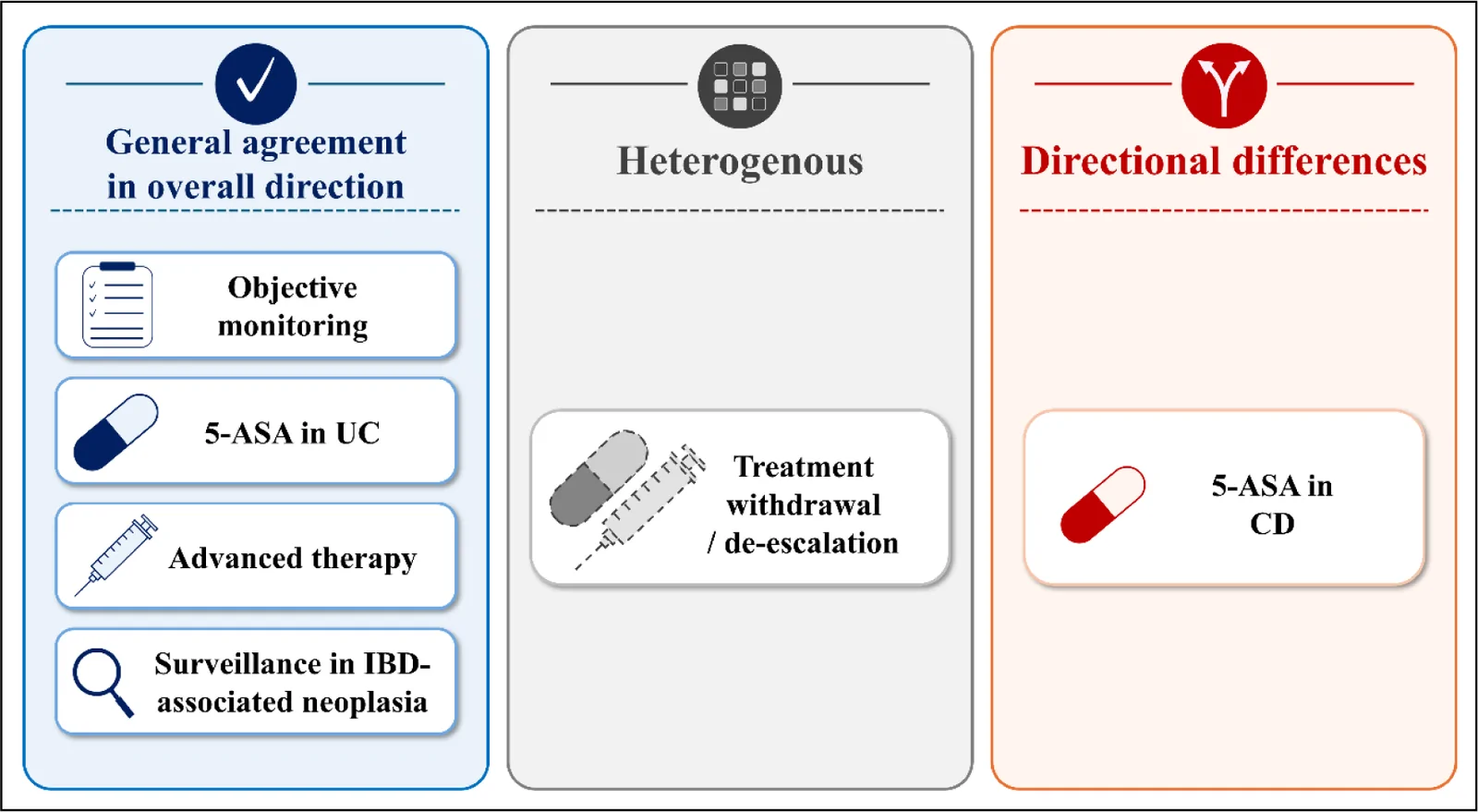
Interpretive summary of concordance, divergence, and heterogeneity across key IBD guideline domains. This figure summarizes how the principal comparison domains in Chapter 5 are best interpreted across the included guideline sets. It distinguishes general agreement in overall direction, directional differences, and heterogeneous domains with uneven comparability across source documents. Objective monitoring, 5-ASA in UC, the broader incorporation of advanced therapies, and surveillance in IBD-associated colorectal neoplasia are shown as areas of general agreement in overall direction; 5-ASA in CD as the clearest area of directional differences; and treatment withdrawal/de-escalation as a heterogeneous domain. Abbreviations: 5-ASA, 5-aminosalicylic acid; CD, Crohn's disease; IBD, inflammatory bowel disease; UC1 , ulcerative colitis

**Figure 4:**
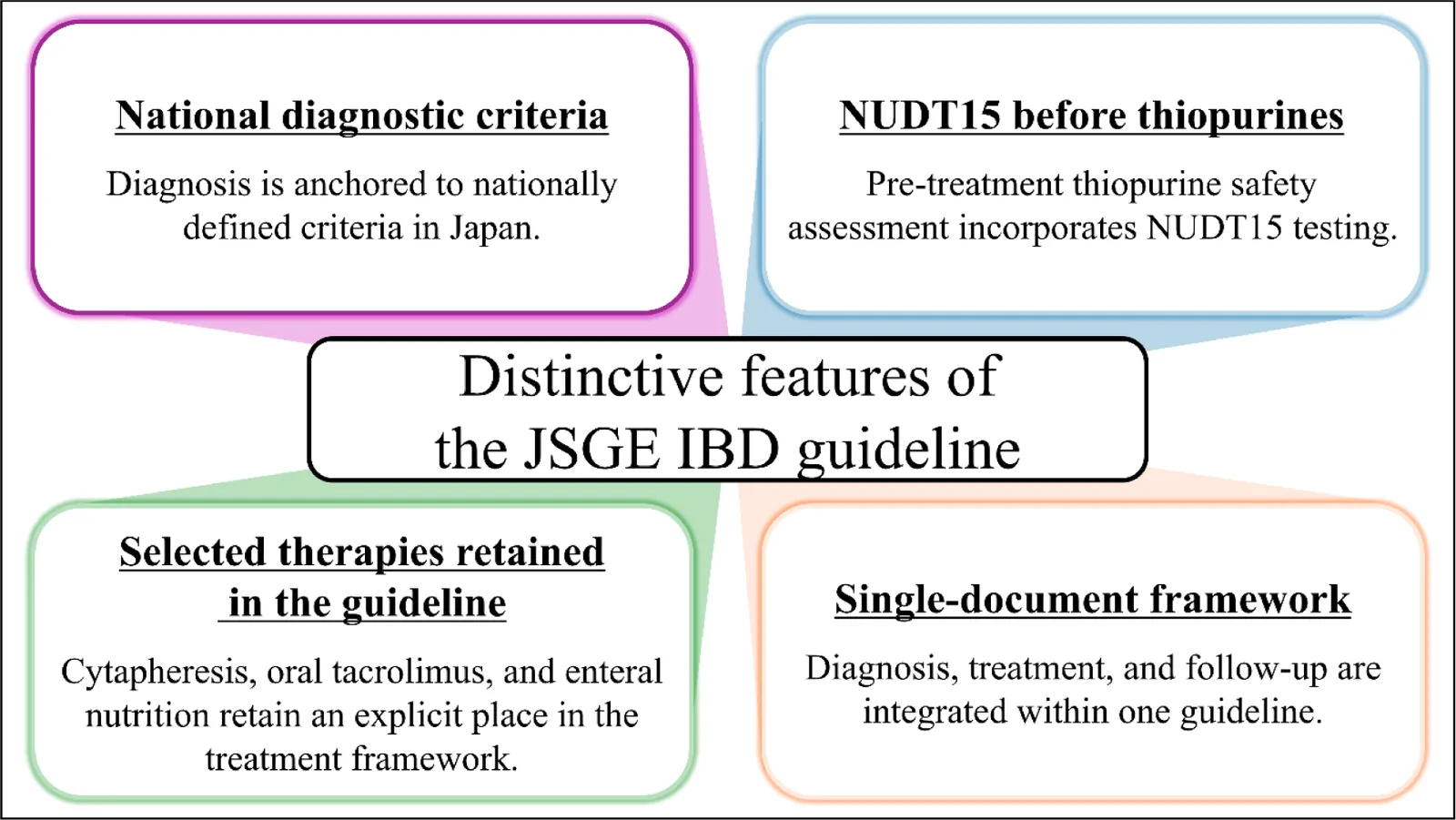
Distinctive features of the JSGE IBD guideline in this comparison. This figure highlights four features emphasized in Chapter 6: diagnosis anchored to nationally defined criteria, NUDT15-based pre-treatment thiopurine safety assessment, continued visibility of cytapheresis and oral tacrolimus, and integration of diagnosis, treatment, and follow-up within a single guideline document. These features reflect a specific clinical and institutional context rather than a simple hierarchy between the Japanese guideline and more modular international systems. Abbreviations: IBD, inflammatory bowel disease; JSGE, Japanese Society of Gastroenterology; NUDT15, nucleoside diphosphate-linked moiety X-type motif 15
